# Reduced Cerebral Oxygen Content in the DG and SVZ In Situ Promotes Neurogenesis in the Adult Rat Brain In Vivo

**DOI:** 10.1371/journal.pone.0140035

**Published:** 2015-10-14

**Authors:** Kuan Zhang, Yanzhao Zhou, Tong Zhao, Liying Wu, Xin Huang, Kuiwu Wu, Lun Xu, Dahu Li, Shuhong Liu, Yongqi Zhao, Ming Fan, Lingling Zhu

**Affiliations:** 1 Department of Brain Protection and Plasticity, Institute of Basic Medical Sciences, Beijing, China; 2 Co-innovation Center of Neuroregeneration, Nantong University, Nantong, China; 3 Beijing Institute for Brain Disorders, 10 Xitoutiao, You Anmen, Fengtai District, Beijing, P.R. China; 4 Brain Research Center, College of Basic Medical Sciences, Third Military Medical University, Chongqing, China; University of Tennessee Health Science Center, UNITED STATES

## Abstract

Neurogenesis in the adult brain occurs mainly within two neurogenic structures, the dentate gyrus (DG) of the hippocampus and the sub-ventricular zone (SVZ) of the forebrain. It has been reported that mild hypoxia promoted the proliferation of Neural Stem Cells (NSCs)*in vitro*. Our previous study further demonstrated that an external hypoxic environment stimulated neurogenesis in the adult rat brain *in vivo*. However, it remains unknown how external hypoxic environments affect the oxygen content in the brain and result in neurogenesis. Here we use an optical fiber luminescent oxygen sensor to detect the oxygen content in the adult rat brain *in situ* under normoxia and hypoxia. We found that the distribution of oxygen in cerebral regions is spatiotemporally heterogeneous. The Po_2_ values in the ventricles (45∼50 Torr) and DG (approximately 10 Torr) were much higher than those of other parts of the brain, such as the cortex and thalamus (approximately 2 Torr). Interestingly, our *in vivo* studies showed that an external hypoxic environment could change the intrinsic oxygen content in brain tissues, notably reducing oxygen levels in both the DG and SVZ, the major sites of adult neurogenesis. Furthermore, the hypoxic environment also increased the expression of HIF-1α and VEGF, two factors that have been reported to regulate neurogenesis, within the DG and SVZ. Thus, we have demonstrated that reducing the oxygen content of the external environment decreased Po_2_ levels in the DG and SVZ. This reduced oxygen level in the DG and SVZ might be the main mechanism triggering neurogenesis in the adult brain. More importantly, we speculate that varying oxygen levels may be the physiological basis of the regionally restricted neurogenesis in the adult brain.

## Introduction

An increasing number of studies have recently demonstrated that neural stem cells favor mild hypoxia and that hypoxia is a potent stimulator of the expansion of neural stem cells in vitro [1–3]. Hypoxia can also promote the in vitro differentiation of neural stem cells into neurons, particularly dopaminergic neurons [1, 4]. In the adult brain, neural stem cells have been found at high densities in the forebrain subventricular zone (SVZ) and the dentate gyrus (DG) of the hippocampus, and increased neurogenesis in vivo was reported to be involved in the regulation of learning, memory, brain injury repair, and anti-depression [5–9]. It has been previously reported that hypoxia treatment promote the angiogenesis [10], gliogenesis [11,12], and neurogenesis [13, 14] in vivo. Our previous studies also showed that intermittent application of a hypoxic environment in vivo significantly promoted neurogenesis in the rat brain [2] and further demonstrated that this periodic hypoxia treatment could improve the disorder of depression by increasing neurogenesis in the hippocampus [15]. In addition, hypoxia exposure has also been found can induce HIF-1αexpression in neurogenic regions of the adult rodent brain, including the dentate gyrus (DG) and subventricular zone (SVZ) [10,15,16]. However, almost all the existing data related to cerebral oxygen tension during hypoxia exposure were indirect [17–19]. It remains unclear whether external hypoxia affects the actual oxygen content in the SVZ and DG in the brain as a means of promoting neurogenesis.

Actual in vivo oxygen concentrations provide a significant index of tissue metabolic levels in different physiological or pathological states. Brain function varies from region to region; metabolic activity and oxygen demands likewise differ across the brain. The oxygen supply must be precisely controlled in the brain in response to local demands induced by metabolic activity [20, 21]. Using different oxygen probes, the actual oxygen content has been reported to range from 1 to 5 percent and even lower in some regions of the brain [22]. Here, we employed an optical fiber luminescent oxygen sensor (MICROX TX3, fiber-optic oxygen meter) to directly measure the oxygen content in the entire cerebral area as well as real-time temporal changes in Po_2_ in various cerebral regions, particularly in the SVZ and DG, where neurogenesis occurs in response to reduced external oxygen content.

In the present study, we reveal that as ambient oxygen levels declined, Po_2_ values in the DG and SVZ also decreased. The reduced oxygen partial pressure in the DG and SVZ could be the main cause triggering neurogenesis in the adult brain. More importantly, we speculate that differences in oxygen levels may represent the physiological basis restricting neurogenesis to certain areas within the adult brain.

## Materials and Methods

### Ethics statement

Our experiments were approved by the Experimental Animal Center at the Academy of Military Medical Sciences. All procedures in our experiments were performed strictly in accordance with the guidelines of the National Institutes of Health (NIH) for animal care. Every effort was made to minimize suffering and the number of animals used. Sodium pentobarbital was used to anesthetize the animals.

### Experimental animals

Adult male SD rats (weighing 250~300 g or 180~200 g) maintained in the Academy of Military Medical Sciences’ animal house were used for all experiments. Animals were allowed unlimited access to standard laboratory chow and water and were maintained at a constant temperature (24°C±2°C) with a 12-h light–dark cycle.

### Stereotaxic surgery for intracerebral implantation of the fiber-optic oxygen microprobe

Surgery was performed at room temperature under general anesthesia with sodium pentobarbital (60 mg/kg, i.p.). During surgery, the animals’ heads were placed in a stereotaxic apparatus, and their body temperature was maintained at 35–37°C with a fluid-filled heating pad. A skin incision was made on the midline of the head, and the working area was exposed with a retractor. The soft tissues were scraped with a surgery blade and cleaned with sterilized cotton-tipped applicators. After cleaning the skull tissues, one hole (2.0 mm in diameter) was made with a sterilized trephine operated by a drill at each of the following coordinates: (*AP*, 0 mm; *LR*, 1.3 mm; *D*, -1.5 mm) in the cortex; (*AP*, 0 mm; *LR*, 1.3 mm; *D*, -4.0 mm) in the lateral ventricle; (*AP*, 3.6 mm; *LR*, 2.0 mm; *D*, -2.5 mm) in the CA1 of the hippocampus; (*AP*, 3.6 mm; *LR*, 2.0 mm; *D*, -2.8 mm) in the DG of the hippocampus; (*AP*, 3.6 mm; *LR*, 2.0 mm; *D*, -3.1 mm) in the hilus of the hippocampus; (*AP*, 3.6 mm; *LR*, 2.0 mm; *D*, -4.0 mm) in the third ventricle; (*AP*, 3.6 mm; *LR*, 2.0 mm; *D*, -5.5 mm) in the thalamus; and (*AP*, 0 mm; *LR*, 3.0 mm; *D*, -4.0 mm) in the striatum. The coordinate system we used in this experiment was defined as follows: the anterior fontanel (A-F) on the cranium was taken as the zero point (*AP*, 0.0 mm; *LR*, 0.0 mm; *D*, 0.0 mm) of the whole coordinate system; the surface of the cranium was taken as the zero point for the level direction (D, 0.0 mm); and the midline of the cranium was taken as the zero point for the lateral direction (LR, 0.0 mm). Continuous monitoring of arterial oxygen saturation (SaO_2_), respiration rate (RR), heart rate (HR) and body temperature (T) assured maintenance of basic physiological parameters. The mean values of the physiological measurements during the experiments were as follows: SaO_2_ (98.1±0.85%); RR (65.0±4.97 breaths/min); HR (305.8±12.3 beats/min); T (36.7±0.46°C); n = 5.

### Calibration of the optical fiber luminescent oxygen sensors

The model of the optical fiber luminescent oxygen sensors used in our experiments is ‘IMP-PSt1-L5-LIC1-BGF3-TF-YOP; 140 μm’ (Precision Sensing GmbH, http://www.presens.de; Germany). The chemical materials quenched by oxygen are polycyclic aromatic hydrocarbons, transition metal complexes of Ru(II), Os(II) and Rh(II), and phosphorescent porphyrins containing Pt(II) or Pd(II) as the central atom. A PT 1000 temperature sensor is combined with the Microx TX3 to record temperature variations, which are compensated using the Microx TX3 software. To measure the Po_2_, the optical fiber luminescent oxygen sensors were calibrated before and after each experiment in a freshly prepared 1% Na_2_SO_3_ solution used to make oxygen-free water (calibration solution 0) and in a vessel filled with wet cotton wool that represented water vapor-saturated air (calibration standard 100). The daily barometric pressure was 1007 hPa. During each calibration, the microprobe was immersed in the standard solution or air until the ‘phase angle’ used for calibration became constant. The accuracy of the microprobe is ±1% at 100% air-saturation and ±0.15% at 1% air-saturation’ (Instruction Manual, MICROX TX3, Fiber-optic oxygen meter, www.presens.de; Germany).

### Real-time measurement of the oxygen distribution in rat brain tissues

**Fig 1:**
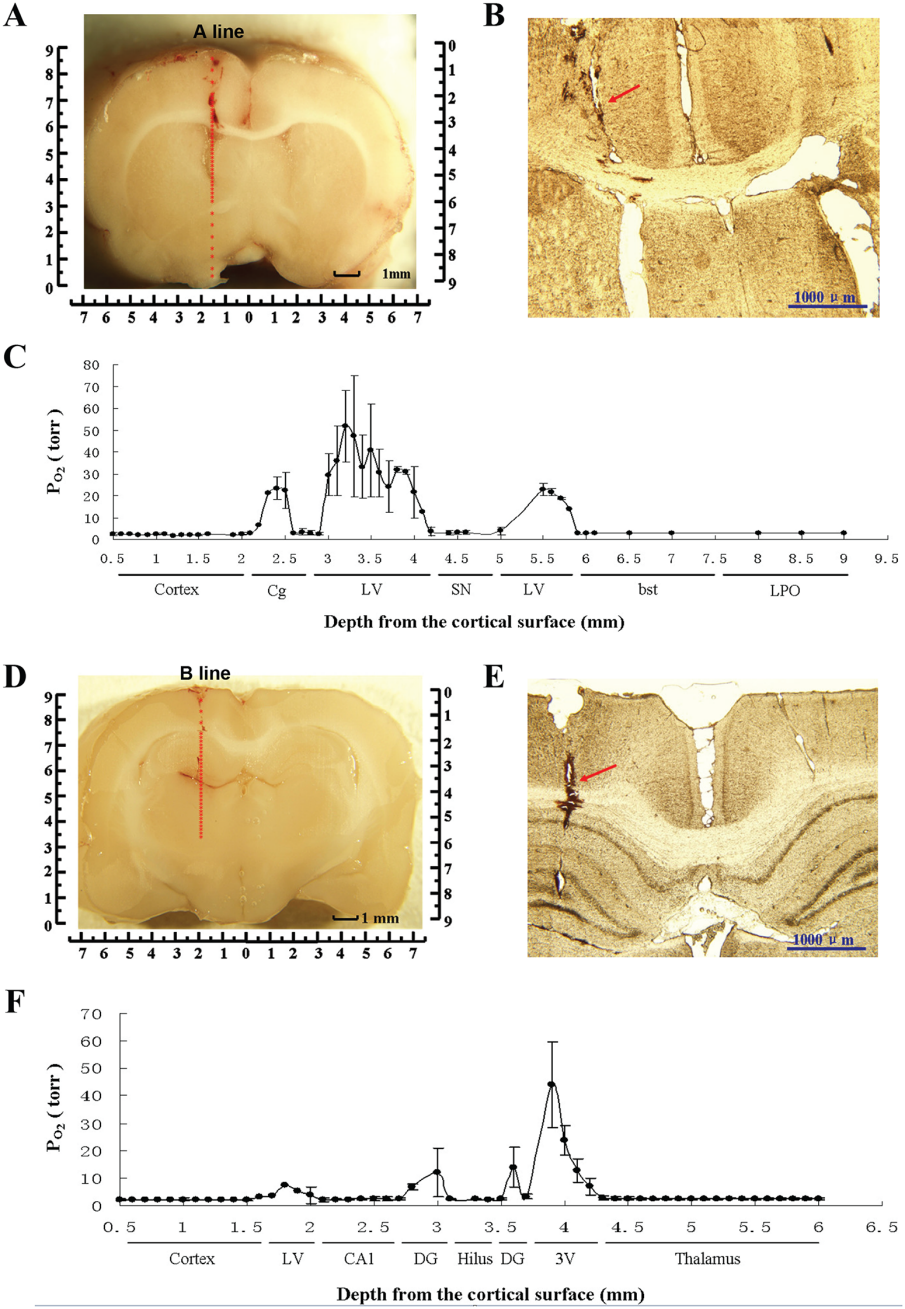
Spatial distribution of oxygen in brain tissues in vivo. (A) Sample points on the A line. The red dots indicate the sample points separated by 0.1 mm along the A line (*AP*, 0 mm; *LR*, 1.3 mm; *D*, 0.0~-9.0 mm). (B) The trail of microprobes on the A line. The red arrow points to the trail left by the microprobe along the A line in the brain tissue. (C) Po_2_ values in different encephalic regions along the A line (n = 6). Po_2_ values in the Cg and LV are much higher than those in other regions of the brain. (D) Sample points on the B line. The red dots indicate the sample points separated by 0.1 mm along the B line (*AP*, 3.6 mm; *LR*, 2.0 mm; *D*, 0.0~-6.0 mm). (E) The trail of microprobes on the B line. The red arrow points to the trail left by the microprobe along the B line in the brain. (F) Po_2_ values in different encephalic regions along the B line (n = 6). Po_2_ values in the DG, LV and 3 V are much higher than those in other regions of the brain.

The entire process of microprobe insertion and the subsequent measurements were performed under general anesthesia with sodium pentobarbital (60 mg/kg, i.p.). To measure the spatial distribution of oxygen in brain tissues, two vertical lines across the coronal planes of the main encephalic regions were selected: A line (*AP*, 0 mm; *LR*, 1.3 mm; *D*, 0.0∼-9.0 mm) and B line (*AP*, 3.6 mm; *LR*, 2.0 mm; *D*, 0.0∼-6.0 mm) (Fig 1A and 1D). The calibrated oxygen microprobe was stereotaxically positioned above the A line (*AP*, 0 mm; *LR*, 1.3 mm; *D*, 0.0∼-9.0 mm) or B line (*AP*, 3.6 mm; *LR*, 2.0 mm; *D*, 0.0∼-6.0 mm) and slowly inserted from the cortical surface through the corresponding hole drilled earlier. The measurement was started at 0.5 mm under the cortical surface and ended at 9.0 mm or 6.0 mm beneath the cortical surface in the A and B line tests (Fig 1A and 1D). Po_2_ values were recorded at each sample point separated by 0.1 mm along the A or B line (Fig 1A and 1D) using a data acquisition program matched with the interface (Microx T3, http://www.presens.de). Six rats each were used to measure Po_2_ values along A and B lines.

### Real-time measurement of Po_2_ in the brains of living animals under a hypoxic environment

To investigate whether a hypoxic environment can influence Po_2_ in living animals, we moved the platform used to test the Po_2_ to a large complex hypoxic capsule, which can model the hypoxic environment encountered at high altitudes. The real-time temperature, humidity, oxygen saturation, altitude and the speed of lifting or descending were monitored. To explore how Po_2_ changes in the brains of living animals under different environments, we first placed the oxygen sensor in different areas of the rat brain. The dentate gyrus of the hippocampus (*AP*, 3.6 mm; *LR*, 2.0 mm; *D*, -2.8 mm) and the sub-ventricular zone (*AP*, 0 mm; *LR*, 1.3 mm; *D*, -4.0 mm) were chosen for their neurogenic capacity. Beginning with the Po_2_ at sea level, the altitude the capsule models increased at a speed of 5 m/s. At the same time, we also observed how the Po_2_ in the brain changed over time at different levels of external oxygen content. Here, the altitude was maintained at a stable level (2000 or 3000 meters), and the Po_2_ in the brain was recorded over a period of 30–60 min.

To investigate normal physiological parameters under these complex environments, a small animal pulse oximeter (Life Sciences Co., Australia) and laser Doppler flowmetry (Biopac Co., USA) were used. To test the heart rate, breath rate and oxygen saturation, animals were anesthetized, and the neck was clamped by the clip connected to the pulse oximeter (Life Sciences Co., Australia). A computer connected to the instrument recorded real-time data from the animals. To detect changes in regional cerebral blood flow, an operation was performed as described [23], and the probe was fixed on the surface of the endocranium, allowing real-time relative cerebral blood flow patterns to be recorded.

### Histological analysis of the locations of the optical fiber luminescent oxygen sensors

After the Po_2_ recordings were completed, the anesthetized animals were immediately perfused with ice-cold phosphate-buffered saline (PBS) followed by 4% paraformaldehyde via the left ventricle of the heart. The whole brain was removed and post-fixed in 4% paraformaldehyde in 15% sucrose for 24 h followed by 30% sucrose for dehydration for 12 h. Consecutive coronal sections 40 μm in thickness were prepared using Frozen Sectioning techniques (Thermo, USA). The sections at the corresponding coordinates were examined (Fig 1B and 1E).

### Pimonidazole immunohistochemistry

Male Rats were given a intraperitoneal injection of pimonidazole (hypoxyprobe, USA) solution at a dosage of 60mg/kg. 3 hours after the administration, rats were sacrificed and perfused. Consecutive brain sections were collected, rinsed in PBST (0.3%) for 1h and blocked in 5% BSA for 2h at room temperature. Then, sections were incubated with a mouse anti-pimonidazole primary antibody (1:50, hypoxyprobe, USA) at room temperature for 2h. The slides were then washed in 0.1 M phosphate buffer 3 times and incubated with biotin-conjugated Donkey Anti-mouse secondary antibody for 2h. Using an ABC kit (Vector Laboratories, USA), pimonidazole-positive cells were visualized and captured with a microscope (Olympus, Japan).

### Intermittent hypoxia procedure

Animals underwent intermittent hypoxia as described with minor modifications [2]. Briefly, male SD rats (weight: 180~200 g) were randomly divided into two groups. Group one animals were subjected to a hypoxic environment equivalent to an altitude of 3000 m in a transparent chamber for 4 h per day for 2 consecutive weeks. Group two animals were kept under the same conditions without an increase in altitude. After the intermittent hypoxia procedure, animals were placed back into their home cages and had free access to food and water throughout the experiment. The number of animals used in each group was 8.

### BrdU administration and brain preparation

Rats in both groups received BrdU injections as previously described [2]. Briefly, BrdU (Sigma), 50 mg/kg in 0.9% saline water, was administered once daily for 3 consecutive days after intermittent hypoxia, and then the rats were perfused with 4% paraformaldehyde. Brains were isolated and sectioned 24 hours after the last BrdU injection. The sections were then processed for immunohistochemistry.

### Immunohistochemistry and cell counting

To detect BrdU-positive cells, the DNA was denatured by 2N HCl, and the sections were incubated with a mouse anti-BrdU primary antibody (Thermo, 1:1000) for 48 h at 4°C. The slides were then washed in 0.1 M phosphate buffer 3 times and incubated with biotinylated goat anti-mouse IgG (Invitrogen, 1:3000) at 4°C overnight. Using an ABC kit (Vector Laboratories, USA), BrdU-positive cells were visualized and counted blindly with a microscope (Olympus, Japan).

### Western blot assay

Rats were sacrificed immediately after the intermittent hypoxia procedure. Brains were removed, and hippocampal and subventricular zone tissues were dissected out and homogenized on ice in a RIPA lysis buffer containing a proteinase inhibitor cocktail (Roche). The samples were centrifuged at 12000 rpm at 4°C for 30 min. Supernatants were kept at -80°C for immunoblotting.

After determining protein concentrations, equal amounts of protein per sample were subjected to electrophoresis and transferred to PVDF membranes (Roche, UK). The membranes were blocked for 2 hours in non-fat milk, and primary antibodies (mouse anti-HIF-1α, Novus, 1:1000; mouse anti-β actin, Sigma, 1:10000; mouse anti-VEGF, Abcam, 1:500) were applied overnight at 4°C. The membranes were washed 3 times in TBST and incubated with a secondary antibody (HRP-conjugated goat-anti-mouse, MBL, 1:2000) at room temperature for 2 hours. The membranes were then subjected to ECL detection (Bio-Rad, USA), and protein bands were quantified with Image J software.

### qRT-PCR analyses

Male SD rats (160-180g) were subjected to 3000m intermittent hypoxia treatment (4h/day) for 2 weeks. Then, the animals were sacrificed. The hippocampus (HP) and subventricular zone (SVZ) were harvested on ice. mRNA were extracted with trizol (ambition,USA) and reverse translated into cDNA with a reverse translate kit (Takara, Japan). Lef-1 and Tcf-1 mRNA expression were evaluated using qRT-PCR method. Primers for Lef-1, Tcf-1 and β-actin are as follows: LEF-1: Forward: 5’-ccccgaagaggagggcgact-3’, Reverse: 5’-tccgaccacctcatgccgtt-3’; TCF-1: Forward: 5’-aagatgacacggatgacgatgg-3’, Reverse: 5’-tgttgaggtgctgggacagg-3’;β-actin: Forward: 5’-ccagttcgccatggatgac-3’; Reverse: 5’-atgccggagccgttgtc-3’.

### Statistical analysis

The data are presented as the means ± standard errors (SE) in qRT-PCR, and means ±standard deviation (SD) in all the other results. An independent-sample t test was used for statistical analyses throughout the investigation.

## Results

### Oxygen distribution in different encephalic regions

The Po_2_ levels in different encephalic regions were recorded along two different lines in the brain (n = 6 rats per line; Fig 1A–1F, respectively). The A line (Fig 1A) included the cortex, cingulum (Cg), lateral ventricle (LV), septal nucleus (SN), bed nucleus of the stria terminalis (bst), and lateral preotic area (LPO) (Fig 1C). The B Line (Fig 1D) included the cortex, lateral ventricle (LV), CA1, dentate gyrus (DG), hilus, third ventricle (3 V), and thalamus (Fig 1F). The data showed that the spatial distribution of oxygen in brain tissues was heterogeneous. The Po_2_ values in the ventricles (up to 45∼50 Torr) and DG (approximately 10 Torr) were much higher than those recorded in other parts of the brain, such as the cortex and thalamus (approximately 2 Torr) (Fig 1C and 1F).

After completing the above measurements, we confirmed the locations of the probes in the brain. Because almost all sample points in the experiments fell on the A and B lines, we analyzed the tissues on the A line (*AP*, 0 mm; *LR*, 1.3 mm; *D*, 0.0∼-9.0 mm) and B line (*AP*, 3.6 mm; *LR*, 2.0 mm; *D*, 0.0∼-6.0 mm) to determine whether the oxygen microprobes had been placed on the correct route. Representative locations of the fiber-optic oxygen microprobes in brain tissues are shown in Fig 1B and 1E. The red points in Fig 1A and 1D respectively indicate sample points on the A and B lines. The red arrows in Fig 1B and 1E point to the trails left by the microprobes in the brain tissue. Through these trails, we can see that the microprobes were placed on the correct pathway along the A and B lines.

Pimonidazole is a hypoxia marker and has been used to label the hypoxic regions of brain [17–19]. Here, pimonidazole staining were next used to confirm our current direct measurement with optical fiber luminescent oxygen sensors. The pimonidazole staining indicated that the oxygen levels were very low in the cortex, striatum, thalamus, CA1 and CA3, but relatively high in SVZ, cg and DG (S1 Fig). So these results displayed by the pimonidazole staining were consistent with the data directly acquired by the optical fiber luminescent oxygen sensors.

### Hypoxic environments reduce oxygen levels in both the DG and SVZ in situ

**Fig 2:**
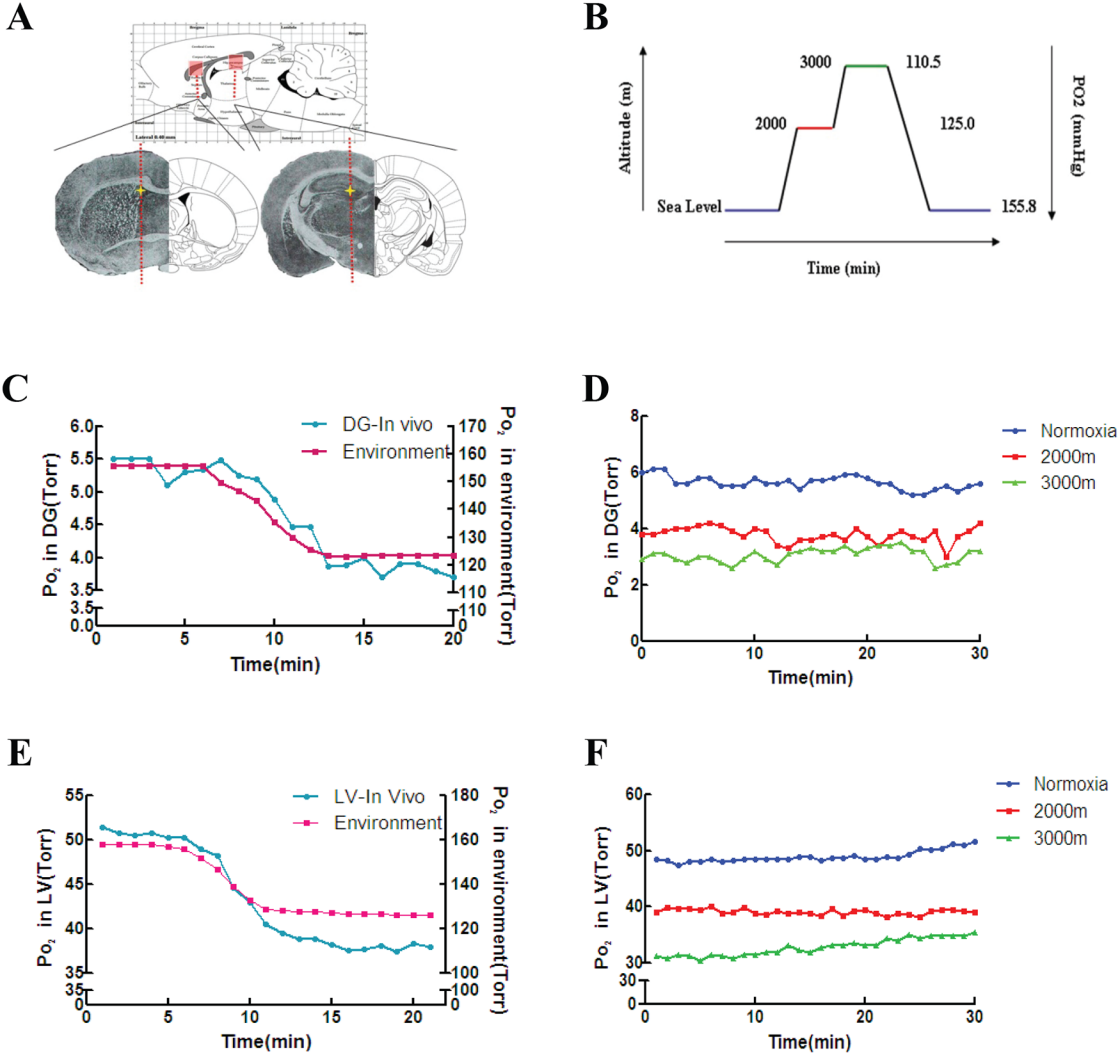
Po_2_ measurements of DG and LV in vivo under normoxia and at altitudes of 2000 and 3000 m. (A) The yellow stars indicate the sites of oxygen measurements in the DG and LV along the A and B lines. (B) Ambient Po_2_ gradually decreases as altitude increases. (C) The Po_2_ in the DG in vivo decreased with the decline in ambient Po_2_. (D) The Po_2_ in the DG was highest under normoxia, then progressively decreased at 2000 and 3000 m. (E) The Po_2_ of the LV in vivo decreased with the decline in ambient Po_2_. (F) The Po_2_ of the LV was highest under normoxia and progressively declined at 2000 and 3000 m.

Neurogenesis in the adult brain is mainly restricted to two specific brain regions: the sub-granular zone (SGZ) of the hippocampus and the sub-ventricular zone (SVZ) of the lateral ventricle [24]. Intermittent hypoxia can stimulate neurogenesis in vivo [2,15]. To investigate whether external hypoxia influences the oxygen content of the neurogenic niches in the adult rat brain, the oxygen content in the DG of the hippocampus (AP: 3.6 LR: 2 D:3) and the lateral ventricle (AP:0 LR:1.3 D:3.1) were measured under different hypoxic conditions that mimicked high altitude environments (Fig 2A).

With the elevation of the altitude, Po_2_ in the ambient environment decreased (Fig 2B). The Po_2_ in the lateral ventricle decreased from 50 to 35 Torr as the ambient Po_2_ decreased from 155.8 Torr (sea level) to 125 Torr (2000 m above sea level) (Fig 2E). The Po2 value in the lateral ventricle stabilized at 50, 40, and 30 Torr when the ambient Po2 was stably maintained at 155.8 Torr (sea level), 125.0 Torr (2000 m) and 110.5 Torr (3000 m), respectively. (Fig 2F). For the DG of the hippocampus, as the ambient Po_2_ decreased from 155.8 Torr to 125 Torr, the Po_2_ in the DG decreased from 5.5 Torr to 3.5 Torr (Fig 2C). When the Po_2_ in the ambient environment was held stable at 155.8 Torr (sea level), 125.0 Torr (2000 m) and 110.5 Torr (3000 m), the Po_2_ in the DG stabilized at 6, 4 and 3 Torr, respectively (Fig 2D). These data indicate that hypoxic environments reduce oxygen levels in both the DG and SVZ in vivo.

### Rat physiological parameters change under hypoxic environments

**Fig 3:**
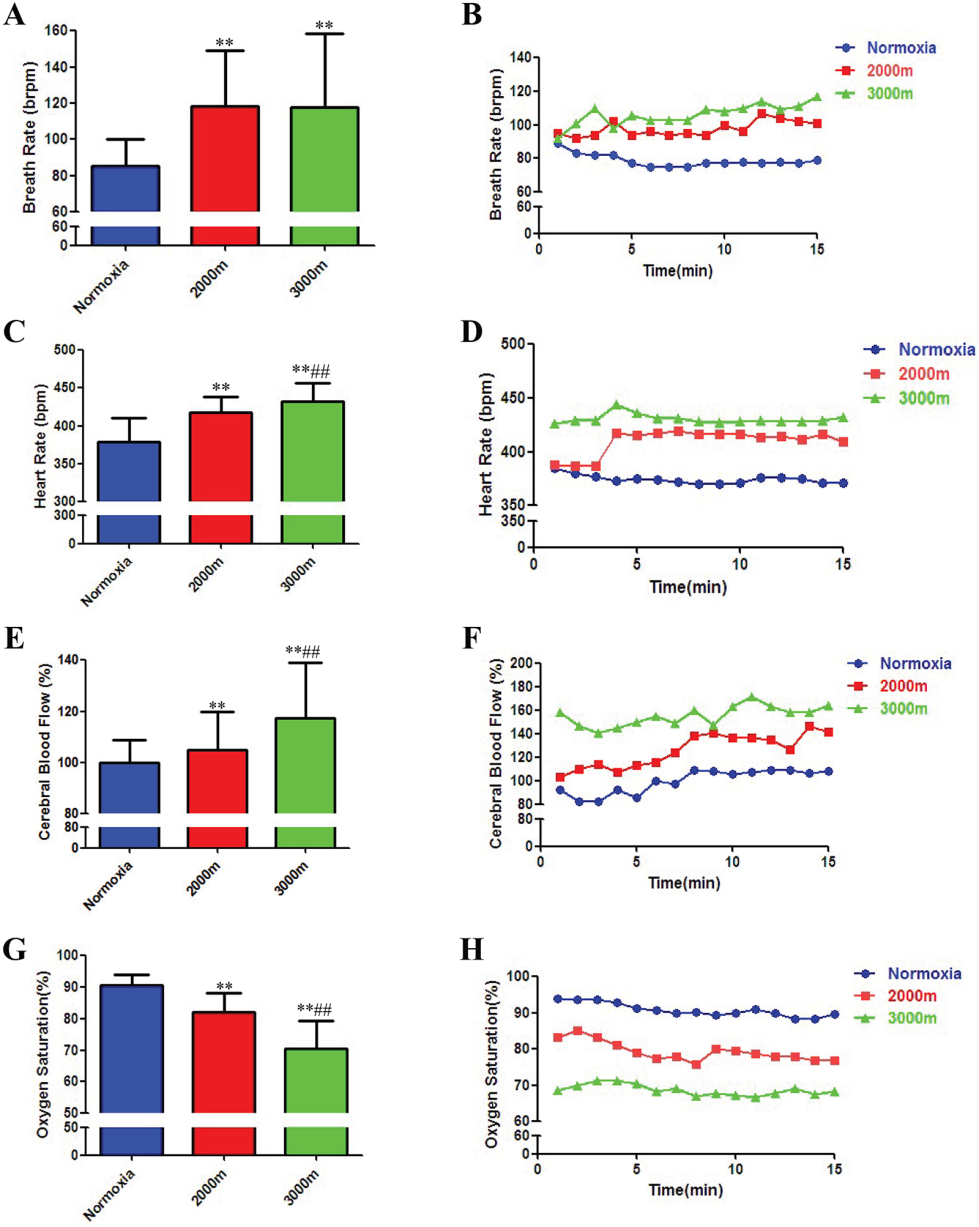
Rat physiological parameters under normoxia and at altitudes of 2000 and 3000 m. (A) Breath rates under different hypoxic environments. (B) Representative plot of real-time breath rate measurements under different hypoxic environments. (C) Heart rates under different hypoxic environments. (D) Representative plot of real-time heart rate measurements under different hypoxic environments. (E) Cerebral blood flow under different hypoxic environments. (F) Representative plot of real-time measurements of cerebral blood flow under different hypoxic environments. (G) Blood oxygen saturation under different hypoxic environments. (H) Representative plot of real-time oxygen saturation measurements under different hypoxic environments. **p<0.01 compared to the normoxia group; ##p<0.01 compared to the 2000 m group.

Rats exhibit adaptive physiological responses under hypoxic environments, including changes in heart rate and breath rate. We therefore simultaneously measured both Po_2_ and general physiological parameters under the hypoxic environment. As the altitude increased from sea level to 2000 and 3000 m, the animals’ breath rate increased from 85 times/min to 118 and 117 times/min, respectively (Fig 3A and 3B), and their heart rate rose from 379 beats/min to 418 and 431 beats/min, respectively (Fig 3C and 3D). At the same time, cerebral blood flow increased by 5% and 17%, respectively (Fig 3E and 3F), and the oxygen saturation decreased from 91% to 82% and 71%, respectively (Fig 3G and 3H). Thus, at high altitudes, the decrease in external oxygen content was accompanied by a decrease in blood oxygen saturation. To compensate, the body adapts to the hypoxic environment by increasing the heart rate, breath rate and cerebral blood flow. These changes observed upon increasing the elevation to 2000 and 3000 m are consistent with previous reports [25,26].

### Hypoxic environments promote the expression of HIF-1α and VEGF in the neurogenic niche

**Fig 4:**
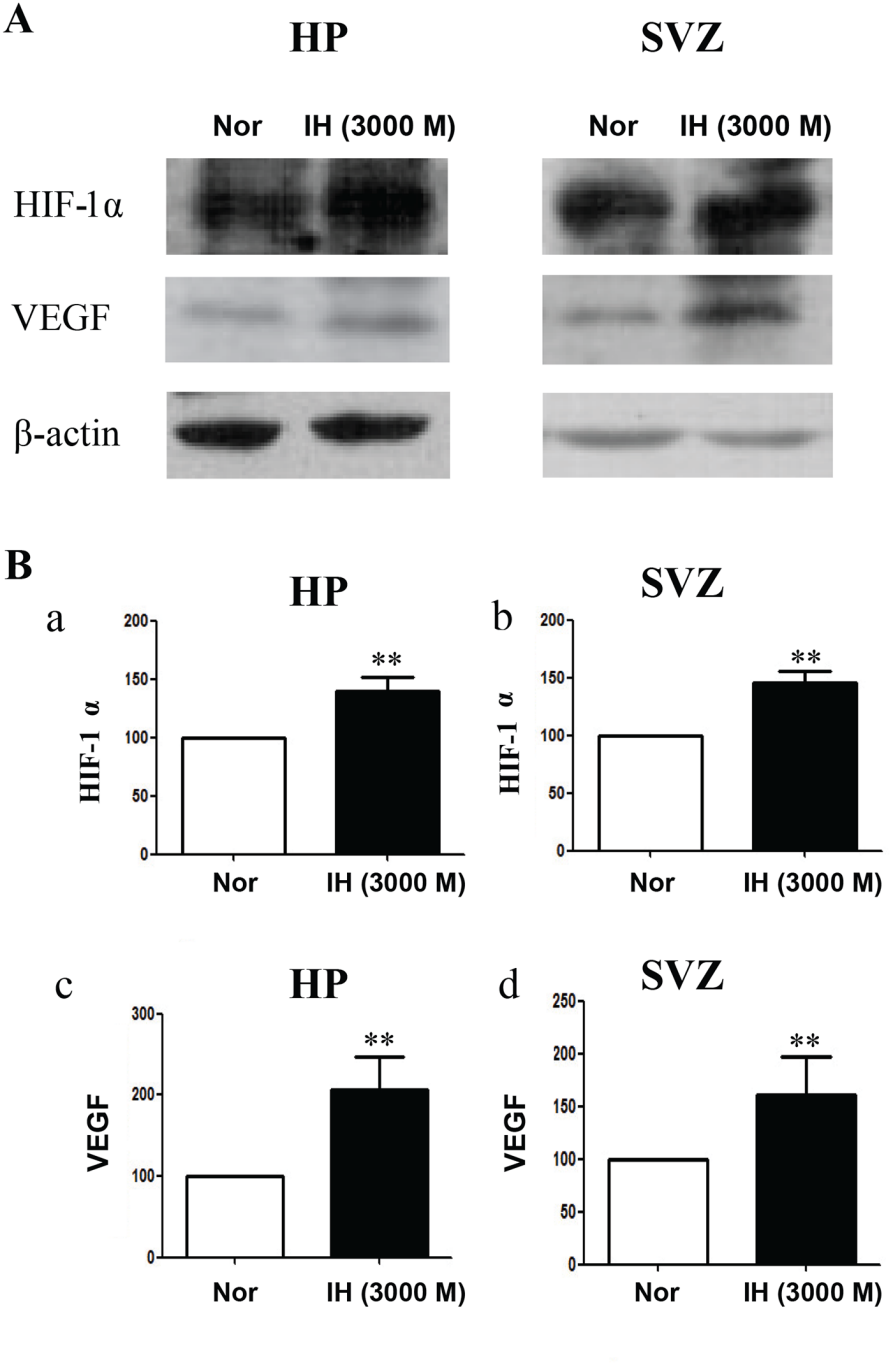
HIF-1α and VEGF expression levels were increased by the 3000 m-intermittent hypoxia (IH) treatment. (A) HIF-1α and VEGF expression in the hippocampus (HP) and sub-ventricular zone (SVZ) was much higher in the intermittent hypoxia (IH) group than in the normoxia group (Nor). (B) Statistical histograms indicating that HIF-1α expression in the HP and SVZ in the IH group was increased 1.39-fold (a) and 1.45-fold (b) relative to the Nor group, respectively; VEGF expression in the HP and SVZ in the IH group was elevated 2.06-fold (c) and 1.06-fold (d) compared with the IH group, respectively. **p<0.01 compared with the Nor group.

HIF-1α is one of the key transcription factors activated in response to hypoxia and regulates more than 100 target genes involved in cell metabolism, survival and apoptosis [27]. VEGF, a HIF-1α target gene, has been reported to mediated hypoxia-induced neurogenesis [6,28,29]. We therefore tested whether the hypoxia treatment altered HIF-1α and VEGF expression in the hippocampus (HP) and SVZ of the brain. We found that HIF-1α expression increased 1.39-fold in the hippocampus and 1.45-fold in the SVZ after the 3000 m intermittent hypoxia (IH) treatment (Fig 4A and 4B). Accordingly, VEGF expression in the IH group also increased 2.06-fold in the HP and 1.60-fold in the SVZ (Fig 4A and 4B) compared with the normoxia (Nor) group. These data indicate that external hypoxia (e.g., 3000 m altitude) can increase the expression of HIF-1α and VEGF in the HP and SVZ in vivo. In addition, consistent with the previous report [30], Wnt/β-catenin signaling pathway can also be activated by external hypoxia treatment (e.g., 3000 m altitude) in neurogenic niche (S2 Fig), which might also be the reason of IH-enhanced neurogenesis.

### Hypoxic environments promotes NSC proliferation in the SGZ and SVZ

**Fig 5:**
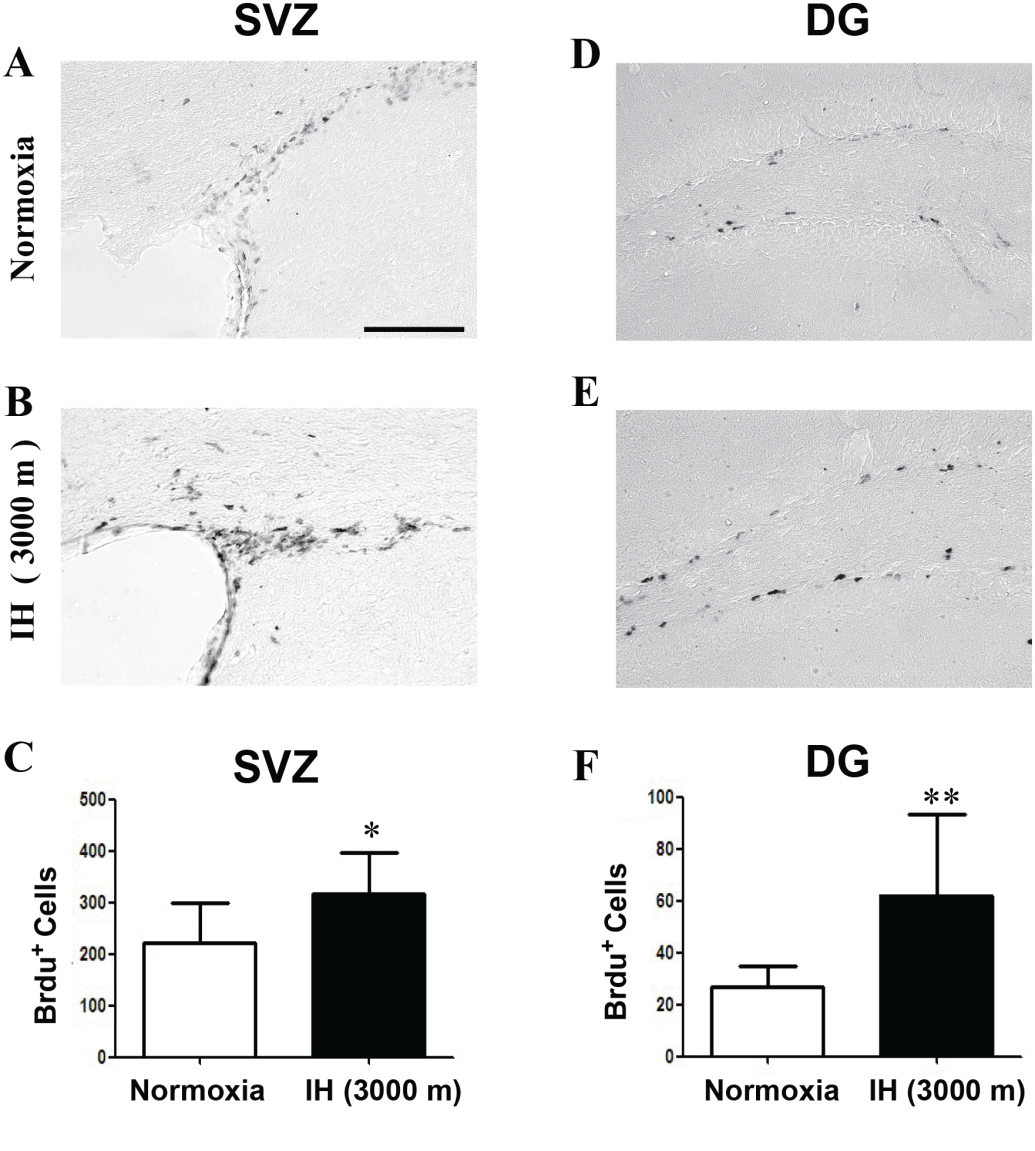
Neurogenesis in the DG and SVZ was increased by the 3000 m-intermittent hypoxia (IH) treatment. IH treatment increased neurogenesis in the SVZ (A, B) and DG (D, E). Statistical histograms showing that the number of BrdU-positive cells increased by 42% in the SVZ (C) and 131% in the DG (F) in the 3000 m-IH group compared with the normoxia group. *p<0.05; **p<0.01; bar = 200 μm; n = 4–5 mice per group.

We next measured neurogenesis by BrdU incorporation in the DG and SVZ after the intermittent hypoxia treatment. In animals exposed to hypoxia, the number of BrdU-positive cells in the SVZ was 42% higher than that of the control group (Fig 5A–5C). Furthermore, the number of BrdU-positive cells in the DG of the hippocampus after hypoxia exposure increased 131% (Fig 5D–5F) compared with the control group. Therefore, consistent with our previous report [2], intermittent simulation of the hypoxia encountered at 3000 m effectively promoted the proliferation of NSCs in both the DG and SVZ *in vivo*.

## Discussion

In the present study, we found that the distribution of Po_2_ in various cerebral regions was not only spatially heterogeneous but also temporally heterogeneous, which might suggest functional and metabolic differences among these areas of the brain. In particular, we found that the Po_2_ in the DG and SVZ, where neurogenesis occurs in the adult, declined simultaneously with the decrease in ambient oxygen. Thus, reduced oxygen partial pressure in the DG and SVZ could represent the main cause triggering neurogenesis in the adult brain.

This study reports continuously measured Po_2_ values along the selected lines and provides quantitative data on Po_2_ at consecutive points across the rat brain in conditions of decreased environmental oxygen content. The data show that the distribution of Po_2_ in the brain is heterogeneous, even in the very small area of one functional unit (Fig 1C and 1F). This heterogeneity may imply different levels of oxidative metabolism in various cerebral regions.

Neurogenesis in the adult brain is regulated by both external and internal factors. Oxygen is one of the key regulators of neurogenesis both in vitro and in vivo. Hypoxia augments the self-renewal of neural stem cells via hypoxia-inducible factor 1α-activated transcription [31,32] and Wnt/β-catenin signaling pathway [30]. Hypoxia also increases the production of neurons from neural stem cells in vitro [3,4]. In addition, moderate hypoxia can promote neurogenesis in the DG and SVZ in vivo [2,15,33]. However, the occurrence of neurogenesis in vivo and its relationship with decreasing oxygen levels within the niche in response to hypoxia have remained largely unknown. Here we used optical fiber luminescent oxygen sensors to investigate Po_2_ in real-time in different cerebral regions. Our data show that a hypoxic environment can cause decreases in Po_2_ in the DG and SVZ. Thus, we provide direct evidence that exposure to a hypoxic environment in vivo induced neurogenesis by regulating the actual oxygen partial pressure in the SVZ and DG of the rat brain. Together, these findings suggest that local differences in oxygen content may serve as the physiological basis for the regionally restricted neurogenesis in the adult brain.

In summary, this study reports the use of optical fiber luminescent oxygen sensors for the real-time measurement of Po_2_ in various cerebral regions. We believe that the application of this method will provide us with novel perspectives for understanding the pathological mechanisms of cerebrovascular diseases and processes related to cerebral oxidative metabolism in special environments. With this innovative method, we have found a physiological basis for how a hypoxic environment promotes neurogenesis in the DG and SVZ. Decreases in regional Po_2_ result in the expression of HIF-1α and its downstream target gene VEGF, and also activate Wnt/β-catenin signaling pathway, which subsequently promote neurogenesis. We propose that intermittent hypoxia treatment causes a decline in Po_2_ in the DG and SVZ, causing the up-regulation of HIF-1α and VEGF expression and enhanced neurogenesis. Understanding the dynamic changes in oxygen content within neurogenic niches in situ may help to provide novel approaches for reactivating adult neurogenesis to treat degenerative brain conditions and cognitive decline.

## Supporting Information

S1 Fig Hypoxic region detected by hypoxic marker, anti-pimonidazole adduct antibody in rat brain. Positive immunoreactivity was detected as brown color of the DAB stain. (A) Section at AP-0mm showed that cortex and striatum were strongly stained with hypoxia marker, and tissues around (lateral ventricle) LV were weakly stained. Scale bar = 3 mm. (B) Weak staining regions were found in subventricular zone (SVZ), corpus callosum (cc) and cingulum (cg). Scale bar = 500μm. (C) Cortex was highly immunoreactive with hypoxia marker. Scale bar = 500μm. (D) Section at AP-3.6 mm showed that cortex, hippocampus and thalamus were strongly stained with hypoxia marker, and tissues around (lateral ventricle) LV were weakly stained. Scale bar = 3 mm. (E) In hippocampus, CA1, CA3 and hilus were strongly stained. However, dentate gyrus (DG) were weakly immunoreactive with hypoxia marker. Scale bar = 500μm. (F) Thalamus was highly immunoreactive with hypoxia marker. Scale bar = 500μm.
(TIF)

S2 Fig Wnt/β-catenin signaling pathway is activated by intermittent hypoxia (IH) in hippocampus (HP) and subventricular zone (SVZ) in mRNA level. Real-time PCR assay for Lef-1 (A and B) and Tcf-1 (C and D) mRNA expression in HP and SVZ after normoxia (Nor) or IH treatment. Lef-1 and Tcf-1 mRNA expressions are increased by IH both in HP and SVZ (*P < 0.05 vs. Nor group; **P<0.01 vs. Nor group; n = 3–6 in each group).
(TIF)
