# Supplementary figures and images for: Reduced Cerebral Oxygen Content in the DG and SVZ In Situ Promotes Neurogenesis in the Adult Rat Brain In Vivo
Source: PLoS One. 2015 Oct 14;10(10):e0140035. doi: 10.1371/journal.pone.0140035 (PMC4605722; doi:10.1371/journal.pone.0140035)

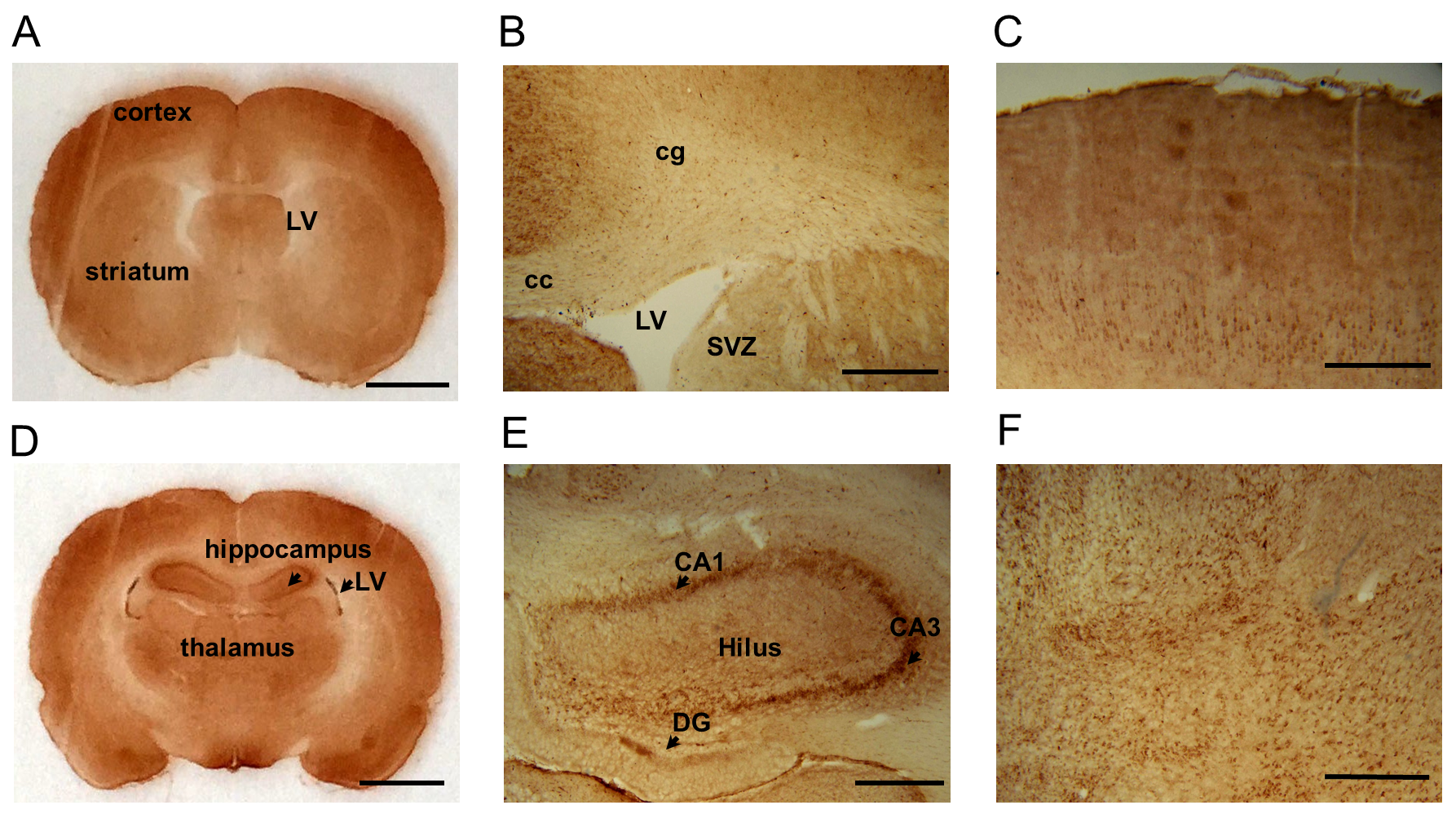

Supplement: S1 Fig — Positive immunoreactivity was detected as brown color of the DAB stain. (A) Section at AP-0mm showed that cortex and striatum were strongly stained with hypoxia marker, and tissues around (lateral ventricle) LV were weakly stained. Scale bar = 3 mm. (B) Weak staining regions were found in subventricular zone (SVZ), corpus callosum (cc) and cingulum (cg). Scale bar = 500μm. (C) Cortex was highly immunoreactive with hypoxia marker. Scale bar = 500μm. (D) Section at AP-3.6 mm showed that cortex, hippocampus and thalamus were strongly stained with hypoxia marker, and tissues around (lateral ventricle) LV were weakly stained. Scale bar = 3 mm. (E) In hippocampus, CA1, CA3 and hilus were strongly stained. However, dentate gyrus (DG) were weakly immunoreactive with hypoxia marker. Scale bar = 500μm. (F) Thalamus was highly immunoreactive with hypoxia marker. Scale bar = 500μm. (TIF) [file pone.0140035.s001.tif]

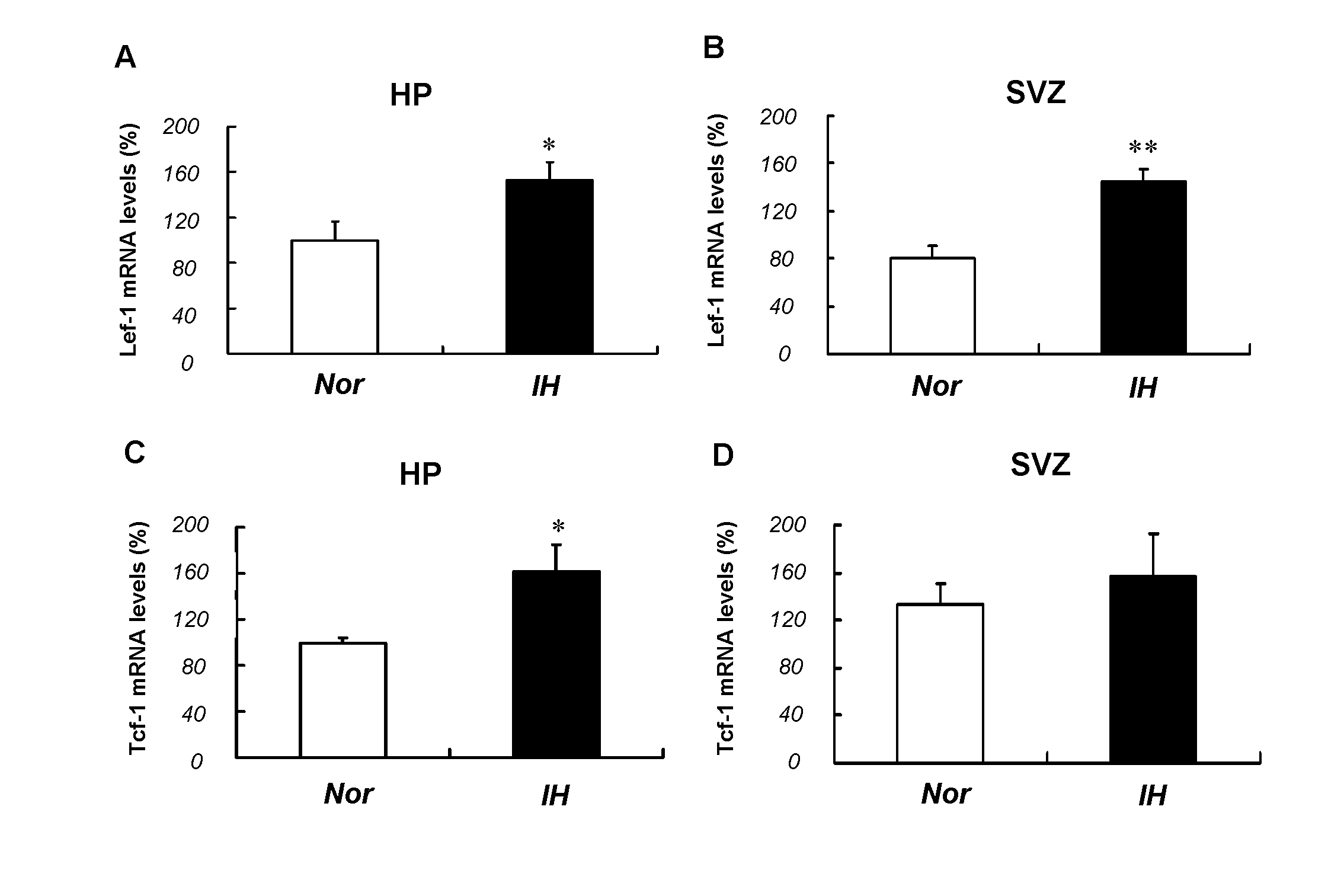

Supplement: S2 Fig — Real-time PCR assay for Lef-1 (A and B) and Tcf-1 (C and D) mRNA expression in HP and SVZ after normoxia (Nor) or IH treatment. Lef-1 and Tcf-1 mRNA expressions are increased by IH both in HP and SVZ (*P < 0.05 vs. Nor group; **P<0.01 vs. Nor group; n = 3–6 in each group). (TIF) [file pone.0140035.s002.tif]
